# Efficacy of an internet and SMS-based integrated smoking cessation and alcohol intervention for smoking cessation in young people: study protocol of a two-arm cluster randomised controlled trial

**DOI:** 10.1186/1471-2458-14-1140

**Published:** 2014-11-05

**Authors:** Severin Haug, Raquel Paz Castro, Andreas Filler, Tobias Kowatsch, Elgar Fleisch, Michael P Schaub

**Affiliations:** Swiss Research Institute for Public Health and Addiction, Zurich University, Konradstrasse 32, 8031 Zurich, Switzerland; Health-IS Lab, Chair of Information Management, ETH Zurich, Weinbergstrasse 56/58, 8092 Zurich, Switzerland; Trier University of Applied Sciences, Environmental Campus Birkenfeld, P.O. Box 1380, 55761 Birkenfeld, Germany; Health-IS Lab, Institute of Technology Management, University of St. Gallen, Dufourstrasse 40a, 9000 St. Gallen, Switzerland

**Keywords:** Tobacco, Smoking cessation, Alcohol, Young people, Internet, Mobile phone

## Abstract

**Background:**

Tobacco smoking prevalence continues to be high, particularly among adolescents and young adults with lower educational levels, and is therefore a serious public health problem. Tobacco smoking and problem drinking often co-occur and relapses after successful smoking cessation are often associated with alcohol use. This study aims at testing the efficacy of an integrated smoking cessation and alcohol intervention by comparing it to a smoking cessation only intervention for young people, delivered via the Internet and mobile phone.

**Methods/Design:**

A two-arm cluster-randomised controlled trial with one follow-up assessment after 6 months will be conducted. Participants in the integrated intervention group will: (1) receive individually tailored web-based feedback on their drinking behaviour based on age and gender norms, (2) receive individually tailored mobile phone text messages to promote drinking within low-risk limits over a 3-month period, (3) receive individually tailored mobile phone text messages to support smoking cessation for 3 months, and (4) be offered the option of registering for a more intensive program that provides strategies for smoking cessation centred around a self-defined quit date. Participants in the smoking cessation only intervention group will only receive components (3) and (4). Study participants will be 1350 students who smoke tobacco daily/occasionally, from vocational schools in Switzerland. Main outcome criteria are 7-day point prevalence smoking abstinence and cigarette consumption assessed at the 6-month follow up.

**Discussion:**

This is the first study testing a fully automated intervention for smoking cessation that simultaneously addresses alcohol use and interrelations between tobacco and alcohol use. The integrated intervention can be easily implemented in various settings and could be used with large groups of young people in a cost-effective way.

**Trial registration:**

Current Controlled Trials ISRCTN02427446 (date of registration: 08th September, 2014).

## Background

Tobacco use is a major contributor to the global burden of disease [1]. Although smoking prevalence among young people in developed countries has been falling over the last two decades, smoking prevalence rates among 15- and 16-year-old adolescents in Europe, at approximately 28%, continues to be a serious problem, particularly among those with lower education levels [2].

Evidence on the effectiveness of smoking cessation interventions for young people is limited. The 2013 Cochrane Review of smoking cessation interventions for those younger than 20 years identified 28 substantial trials, of which only 3 were statistically significant [3]. The authors concluded that there is insufficient evidence to recommend the implementation of any one intervention model and that there continues to be a need for well-designed, adequately powered, randomized controlled trials.

The Internet and mobile phones are extremely popular among adolescents and young adults and are potential media for delivering smoking cessation support to large proportions of the population. In 2012, 95% of 12- to-19-year-old Swiss adolescents owned a mobile phone and 89% used the Internet daily or several times per week. Mobile phone use was the most frequent leisure time activity, while texting was the most frequent activity when using a mobile phone [4].

Expert system technology that provides information based on individual demographic- or smoking-related characteristics can be a viable time- and cost-saving alternative to interpersonal counselling [5]. Short message service (SMS) provides an opportunity for individualized and interactive information delivery that may be accessed easily, independent of time and place. A Cochrane Review on five randomized and quasi-randomized studies revealed overall long-term benefits of mobile phone interventions for smoking cessation among adults; however, a high level of statistical heterogeneity in the pooled results was found [6]. A recent meta-analysis investigating SMS-based health promotion interventions confirmed its efficacy and revealed that tailoring and personalizing messages was significantly associated with greater intervention effectiveness [7].

To date, two randomized controlled trials tested the efficacy of SMS-based programs for smoking cessation in young people aged 16–25 years [8, 9]. To test the *Stop My Smoking USA* program, 164 daily smokers, aged 18–25 years who seriously considered quitting were recruited through an online advertisement and were randomly assigned to either the 6-week intervention program or a matched control group for improving sleep and physical activity [9]. Intervention content was tailored to the participant’s stage within the quitting process. The intervention group received four messages per day during the pre-quitting stage for 2 weeks; in the quitting stage, they received four to nine text messages per day. The intervention significantly affected self-reported quitting rates at 4 weeks, but not at 3 months post-quitting. However, this study was not adequately powered to detect differences in quitting rates between the study groups at follow-up.

To test the efficacy of the *SMS-COACH* program, 755 vocational school students who were smoking regularly were randomly assigned to an intervention group or an assessment-only control group according to clusters based on their class. Within the online assessment, participants of the intervention group received questions that assessed outcome expectancies of smoking cessation, typical situations or circumstances in which cravings for cigarettes occur, and alternative strategies to handle these situations. During the 3-month intervention period, participants received one text message per week to assess smoking-related target behaviours. The stage of change combining smoking status and intention to quit according to the Health Action Process Approach (HAPA) [10] was assessed every alternate week. During the other weeks, the number of cigarettes smoked per day/week was assessed in the ‘pre-intention’ stages; in the ‘intention’ or ‘action’ stage, smokers were asked whether they applied individually selected strategies to cope with craving situations. Two feedback messages per week were tailored to the HAPA stages, baseline data, and weekly SMS assessments. The 7-day smoking abstinence rate at the 6-month follow-up was 12.5% in the intervention group and 9.6% in the control group. This difference was not statistically significant; however, the decrease in the mean number of cigarettes smoked per day from baseline to follow-up was significantly greater in the intervention group than in the control group.

To conclude, both studies on text messaging interventions for smoking cessation in young people showed promising results, however, they did not result in higher abstinence rates, suggesting that improving intervention efficacy is a major challenge. The provision of an integrated smoking cessation and alcohol intervention may be a promising approach for improving its effectiveness. Alcohol consumption and tobacco smoking often co-occur. Further, hazardous drinking is highly prevalent in adolescent and young adult smokers. A study conducted on vocational school students revealed that while 81% of smokers drank hazardously, only 49% of non-smokers drank hazardously [11]. Moreover, drinking alcohol increases cigarette cravings, and relapses after successful smoking cessation are often associated with alcohol consumption [12, 13]. This was also confirmed for adolescent smokers [14], suggesting that drinking alcohol during smoking cessation was strongly associated with the first lapse and subsequent intermittent smoking after quitting.

A pilot study on 41 participants testing an integrated smoking cessation and binge drinking intervention for young adult smokers with regular binge drinking revealed higher tobacco abstinence rates in participants receiving the integrated intervention than in those receiving standard smoking cessation treatment (36% vs. 21% at end of treatment at week 12) [15]. A similar pilot study that included 95 young adults, revealed abstinence rates of 21% vs. 9% at the end of treatment [16]. However, in both pilot studies, the differences between the groups were not statistically significant using the α =5% standard.

In the present study protocol, we describe a cluster-randomised trial comparing the efficacy of an optimized version of the former program *SMS-COACH*, now called *MobileCoach Tobacco (MCT)*, a text message-based smoking cessation program for young adults, to an extended program called *MobileCoach Tobacco + (MCT+),* a combined web- and text messaging-based integrated smoking cessation and alcohol intervention. This is the first study to compare an integrated smoking cessation and alcohol intervention to a smoking cessation only intervention within an adequately powered randomized controlled trial. Moreover, this is the first study to test the efficacy of an automated integrated smoking cessation and alcohol intervention for smoking cessation.

## Methods and design

### Design and hypotheses

**Figure 1:**
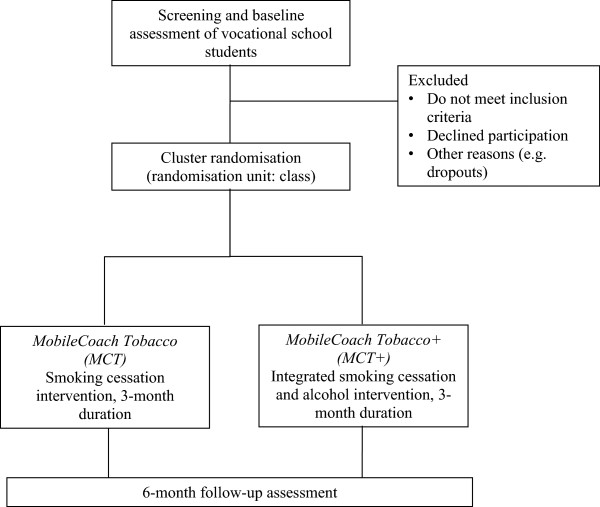
**Study design.**

A two-arm cluster-randomised trial will be conducted to test the efficacy of the *MCT*, a text messaging-based intervention for smoking cessation for young people compared to the *MCT+*, a combined web- and text messaging-based integrated smoking cessation and alcohol intervention. Study participants will be assessed at the baseline and at a 6-month follow-up (Figure 1). Our main hypothesis is that the integrated intervention will be more effective than the smoking cessation only intervention for reducing cigarette consumption and achieving smoking abstinence.

### Participants, setting, and procedure

The interventions will be tested on vocational school students due to the high smoking prevalence in this population (approximately 42% of the Swiss sample were daily or occasional tobacco smokers) [11]. The Cantonal Office for Secondary Education in Zurich and further prevention specialist centres in other Swiss Cantons will request teachers from vocational schools to allow researchers to conduct the Internet- and text messaging-based smoking cessation program with some of their students. Participating teachers will schedule 30 minutes per class for eligibility screening, obtaining informed consent, and collect baseline data.

Study assistants (graduate students of psychology or prevention specialists) will recruit study participants by inviting all vocational school students from a school class to participate in an online health survey during a regular school lesson. Furthermore, they will inform students that some would be invited to participate in a study testing the efficacy of a text messaging intervention for health promotion. To decrease reporting bias, the study assistants will not provide further information about the purpose of the study until the completion of the eligibility screening. This online screening includes data collection on demographics, alcohol consumption, weekly physical activity, smoking status, and mobile phone ownership. Inclusion criteria are: (1) daily or occasional cigarette smoking (at least 4 cigarettes in the preceding month and at least one cigarette during the preceding week) and (2) ownership of a mobile phone.

Subsequently, study assistants will provide the eligible candidates with information about the study aims, interventions, assessments, reimbursement, and data protection, both online and in paper form. Reimbursement of 10 Swiss Francs (~ € 8.00) for participation at the 6-month follow-up assessment will be offered to all study participants. Moreover, participants will be offered 0.5 Swiss Franc (~ € 0.40) for responding to each of the 11 weekly SMS assessments conducted during the program. After obtaining informed consent online, all study participants will be requested to choose a username and provide their mobile phone number. Furthermore, the following smoking related variables will be assessed: intention to quit, daily/weekly cigarette consumption, previous cessation attempts, number of friends who smoke, age at smoking onset, smoking cessation outcome expectancies, situations in which craving for cigarettes occur, alternative strategies to handle these craving situations, and cost per pack of cigarettes. The integrated smoking cessation and alcohol intervention participants will receive additional questions on typical drinking days and times that are necessary for tailoring the intervention content. Computer assisted telephone interviews will be conducted by trained interviewers at the 6-month follow-up assessment.

### Ethical review

The study protocol was approved by the Ethics Committee of the Philosophical Faculty of the University of Zurich, Switzerland (date of approval: 13^th^ August, 2014). The trial will be conducted in compliance with the Helsinki Declaration.

### Randomisation and allocation concealment

To avoid spill over effects within each class, a cluster-randomised controlled trial will be conducted using school classes as a randomisation unit. Due to the heterogeneity of students in various disciplines (e.g. gender or professions), we will use separate randomisation lists for each school (stratified randomisation). Furthermore, to approximate equality of sample sizes in the study groups, we will use block randomisation with computer generated randomly permuted blocks of four cases [17].

The study assistants supervising the baseline assessment in vocational schools will not be informed about group allocation of classes. Group allocation will not be revealed to study participants until they provide informed consent, their username, mobile phone number, and baseline data. Furthermore, the interviewers conducting the computer assisted telephone interviews at the 6-months follow-up will be blinded when assessing the primary and secondary outcome measures.

### Sample size calculation

The effect size estimation was based on the results of a randomized controlled trial testing the efficacy of *SMS-COACH* for smoking cessation among vocational school students that yielded a 7-day point prevalence rate of approximately 12% at the 6-month follow-up in the intervention group [8], and a pilot study testing the efficacy of an integrated smoking cessation and binge drinking intervention that yielded nearly a 1.5-fold increased smoking abstinence rate compared to a standard treatment control [15]. Based on these data, we assumed a 7-day point prevalence abstinence rate of 12% in the smoking cessation only group and 18% in the integrated intervention group at the 6-month follow-up. A sample size of n = 588 in each study group would have 80% power for a Chi-Square Test (α =5%, 2-sided) in order to detect this difference based on G-Power [18]. As vocational school students are nested within school classes, we needed to consider a potential design effect of 1.15 (assumed number of participants per school class: n = 4; intra-cluster correlation coefficient: 0.05 [8]), resulting in an estimated required sample size of n = 675 per study group and a total of N = 1350 study participants.

### Intervention

#### Technological background

The *MCT* and the *MCT +* were developed using the *MobileCoach* system. This modular, web-based system allows an author to easily describe and optimize all parts of an intervention: baseline assessment survey, tailored web-based feedback, intervention rules, text messages with combined content according to group and media, as well as the interplay between these. When using the web-based application interface, technical programming skills are not required for these processes. The open source code of the *MobileCoach* system will be made available on http://mobile-coach.eu by the end of 2014. Two particular aspects of the technical implementation will be discussed here; for further details of the *MobileCoach*, see [19].

The participant–*MobileCoach* system interface is based on SMS text messages. To overcome privacy issues on social networks and the internet in general, SMS offers three advantages: (1) it is safer than typical internet-based technologies (e.g., email, Facebook) because the communication is controlled by one negotiating agent, the mobile operators; (2) the unique phone number allows the system to unambiguously identify each participant by an incoming message, and (3) there is no need for an additional, platform-specific mobile phone application or account. In combination with unique URLs to access audio-visual media content sent with specific text messages, the system can also determine if the content has been viewed by a particular participant.

Another interesting aspect of the *MobileCoach* implementation is the rule-based problem solving approach that builds on the foundations of automata theory [20], rather than the traditional procedural approach. Thus, the system can handle very complex interrelationships involving several variables. Triggered by a small number of possible events (e.g., the participant answers/does not answer a specific text message within a specific time frame) or triggered at a specific time, the system tailors the subsequent intervention steps to the specific user at any time. To do this, the intervention rule-set is applied to the present values of the participants that represent their latest health and behavioural status. With this approach, individually tailored interventions can be modelled with a simple set of rules.

#### Theoretical background

The smoking cessation intervention is primarily based on the HAPA [10]. This health behaviour model suggests a distinction between motivation processes resulting in goal setting (individuals in this stage are called ‘pre-intenders’) and volition processes leading to the actual health behaviour (subdivided into inactive individuals, ‘intenders’; and those who have already adopted these health behaviour, ‘actors’).

Outcome expectancies (pros of smoking cessation and cons of further smoking), risk perception, and perceived self-efficacy in the initial pre-intentional stage are seen as important social-cognitive predictors that promote the intention to act. In the subsequent intentional stage, planning processes are crucial to achieve the desired action. Once an action has been initiated, self-regulatory skills are important to help maintain the health behaviour, e.g. how to cope with craving situations. Additionally, we integrated elements derived from the Social Norms Approach [21] and implementation intentions, ‘if-then’ plans that link situational cues with responses that are effective in attaining a desired outcome [22], into the present intervention.

The alcohol intervention provides normative feedback based on the social norms approach [21], which constitutes the theoretical background of the majority of evidence-based internet interventions to reduce problem drinking in young people [23, 24].

#### Overview of intervention components

After participating in the baseline survey, participants assigned to the *MCT+*, the combined web- and text messaging-based integrated smoking cessation and alcohol intervention will (1) receive individually tailored web-based feedback on their drinking behaviour compared to a reference group representing age and gender norms, (2) receive individually tailored text messages via mobile phones to promote low-risk drinking behaviour for 3-month period, (3) receive individually tailored mobile phone text messages to support smoking cessation for a 3-month period, and (4) be offered the possibility to register for a more intensive program providing strategies for smoking cessation centred around a self-defined quit date.

Participants assigned to *MCT,* the smoking cessation-only intervention, will (1) receive individually tailored mobile phone text messages to support smoking cessation for a 3-month period, and (2) be offered the possibility to register for a more intensive program providing strategies for smoking cessation centred around a self-defined quit date.

The text messages will typically be 150–200 characters long, some of which will include web links to thematically appropriate video clips, pictures, and websites.

#### Web-based feedback on drinking behaviour (MCT+)

Web-based feedback will be provided for participants receiving the *MCT +* immediately after completion of their baseline assessment. The content is based on effective intervention programs developed primarily for college and university students in the USA and Canada [25, 26] modified for the target group of German-speaking adolescents in Switzerland aged 16–20 with varied educational backgrounds. Age- and gender-specific norms for alcohol consumption were derived from a previous study [27] which assessed frequency of risky single occasion drinking, alcohol volume, and the maximum number of drinks consumed on a single occasion among 973 vocational and upper secondary school students in the Canton of Zurich, Switzerland. The web-based feedback includes individually tailored graphical and textual information concerning (1) the number of drinks consumed per week in relation to age and gender-specific reference groups (2) expenditure on drinking, (3) calorific value of consumed alcoholic drinks, and (4) frequency of risky single-occasion drinking in relation to age- and gender-specific reference groups.

#### Text messages stimulating drinking within low-risk limits (MCT+)

Only participants of the *MCT +* group who reported risky single-occasion drinking at the baseline—defined as consuming 5 or more drinks on an occasion for men and 4 or more drinks for women within the previous month—will receive one weekly text message promoting drinking within low-risk limits for a 3 month-period. Every alternative week, this text message will be sent on Saturdays at 7 pm, and during the other weeks, it will be sent on individual’s typical drinking day and time (e.g. Friday at 10 pm).

The text messages will provide information on (1) strategies for drinking within low-risk limits, and (2) the association between smoking and alcohol consumption, particularly on the importance of avoiding alcohol or consuming minimal amounts in order to successfully achieve smoking abstinence for those participants who intend to quit smoking or have already quit. E.g., ‘Hey Susan! Studies show that alcohol consumption clearly leads to an increased desire to smoke. Remember this when you go out the next time and try drinking little or even no alcohol at all. It will be easier for you not to smoke’.

#### Text messages to support smoking cessation (MCT + and MCT)

During the 3-month intervention period, participants in both intervention groups will receive one text message prompt per week that either assesses smoking-related target behaviours or encourages participation in a quiz or a message contest. These prompts can be answered easily by typing a single letter, number, or a sentence using the mobile phone’s reply function. The weekly SMS prompt will be sent at a fixed time each week (Tuesday at 6 pm). The content of the prompt will depend on the individual HAPA stage as well as the number of the intervention week (odd or even).

Smoking-related target behaviour includes the HAPA stage, assessed every 4 weeks by responses to the question: ‘Have you recently smoked cigarettes?’, with the following response options (1) ‘Yes, and I do not intend to quit’ (pre-intender), (2) ‘Yes, but I am considering quitting’ (pre-intender), (3) ‘Yes, but I seriously intend to quit’ (intender), or (4) ‘No, I quit smoking’ (actor). Furthermore, among pre-intenders, we assess the number of cigarettes smoked per day or week (depending on smoking status: daily/occasionally), every 4 weeks, while for intenders and actors, we assess whether the individually chosen strategies to cope with craving situations, assessed within the baseline assessment, were applied, e.g. ‘Did you apply the following strategy recently? When I am at a party, I distract myself from smoking by dancing. Yes (Y) No (N)’.

Participants will receive an immediate feedback SMS after their response on the prompts regarding smoking-related target behaviours, e.g. ‘You can be really proud of yourself! Since the last assessment, you’ve smoked about 4 cigarettes less per day. That means you’re on the right path towards an active and healthy lifestyle.’

Furthermore, 48 hours after this prompt (Thursday at 6 pm) they will receive an additional message tailored to the current HAPA stage and individual data provided at the baseline assessment. Pre-intenders will receive text messages providing information (1) on the risks of smoking, (2) on the benefits of smoking cessation, and (3) regarding methods to improve self-efficacy for smoking cessation. Intenders and actors will receive text messages providing information (1) on how to use individual resources for quitting (e.g. social support), (2) on how to overcome barriers to smoking cessation (e.g. friends who smoke and stress), and (3) methods to improve self-efficacy for smoking cessation. Sample text messages for the different stages and content categories are displayed in Table 1.

**Table 1:** **Sample text messages**

Stage of smoking cessation	Content category	Exemplary text message
Pre-Intention	Risks of smoking	Hi Mary. Smoking and birth control pills aren’t suited to each other. If you smoke while on hormonal birth control, your blood vessels are likely to become constricted and can get blocked. Even for young women, the risk of a heart attack or stroke increases significantly.
	Benefits of smoking cessation	Hi Mike. Even though you aren’t quite convinced yet, we think you should consider quitting; without the constant urge to smoke and buy cigarettes, you’ll feel more independent and confident. Have a look at this video: https://f.mobile-coach.eu/147
	Self-efficacy for smoking cessation	Hi Lizzy. Have you ever wanted to do something that is difficult, such as doing very well at work or similar? Did you succeed? These experiences may also help you become smoke-free!
Intention	Smoking cessation resources	Hi Peter. Sports and exercise can make becoming smoke-free a lot easier. They distract you and result in elevated mood. Maybe you could regularly meet up with a friend to go running, ride bikes, or for other sports activities.
	Barriers to smoking cessation	Hi Alex. Do you have a strong urge to smoke? So does Julia. Both of you are in the same situation. Maybe you could find distractions by spending time together: http://www.juliarauchfrei.at
Action	Self-efficacy for smoking cessation	There are forums online in which smokers and ex-smokers exchange their experiences on smoking cessation. Have a look at http://www.stop-dependance.ch/tabak/forum/

A quiz will be conducted thrice during the intervention period and will include questions concerning: (1) smoking norms (percentage of smokers within the age- and gender-specific reference groups), (2) health consequences of smoking cessation (days until positive health consequences after smoking cessation), and (3) expenditure on cigarettes (money spent for cigarettes per year). Participants will receive immediate individualized feedback on their answer. If they do not respond within 48 hours, they will be sent the correct response.

A contest that requires participants to create a text message to motivate other participants to quit smoking (for intenders) or provide concrete ways to help others quit smoking (for intenders and actors) will be conducted twice during the intervention period. The best text message from each of the two categories, rated weekly by a tobacco cessation expert from the Swiss Research Institute for Public Health and Addiction, will be sent anonymously to participants in the respective category after 48 hours.

#### Optional additional text-messages for smoking cessation (MCT + and MCT)

Participants who intend to stop smoking (intenders and actors according to the HAPA) will be offered the option of receiving additional text-messages for quit-day preparation and relapse prevention. Program participants in these stages will be informed about this option biweekly. After entering a scheduled quit date, the program will provide up to two daily text messages (weeks –1 to +1: two daily text messages; weeks +2 and +3: one daily text message). E.g., ‘Good evening, Marc. Tomorrow’s going to be your first smoke-free day. It would be best for you to throw away all cigarettes, ash trays, and lighters today. Try avoiding boredom tomorrow. Buy some gum for tomorrow in advance.’

### Outcomes

The primary outcome measures assessed at the 6-months follow-up are: (1) 7-day point prevalence smoking abstinence (i.e. not having smoked a puff within the past 7 days), (2) cigarette consumption (number of cigarettes smoked on a typical day for daily smokers, typical number of smoking days per month, and number of cigarettes smoked on a typical smoking day for occasional smokers).

Secondary outcome measures, assessed at the 6-months follow-up include: (1) 30-day point prevalence smoking abstinence (i.e. not having smoked a puff within the past 30 days), (2) stage of change according to the HAPA [10], (3) a quit attempt within the 6-month period, (4) alcohol consumption assessed by a 7-day drinking calendar, for which participants would need to think about a typical week in the month prior to assessment and recall the number of standard drinks they typically consumed for each day.

### Data analyses

We will use regression models to compare the efficacy of the interventions on the different outcome measures. If necessary, we will control for baseline differences by entering additional baseline variables as covariates to the regression models. All analyses will be performed considering the intention-to-treat principle. Given the clustered nature of the data (students in different classes), we will compute robust variance estimators for all regression analyses.

## Discussion

This study protocol presents the design of a cluster randomised controlled trial comparing the smoking cessation efficacy of an integrated smoking cessation and alcohol intervention and a smoking cessation only intervention. Beyond the interventions’ efficacy, we also pay close attention to their retention rates since intervention intensity and complexity is higher in the integrated intervention than in the smoking cessation only intervention. This may result in different dropout rates; however, we do not have a clear directional hypothesis for attrition. Additionally, we will determine the acceptability of the interventions using objective markers, such as the number of views for the video clips that were integrated into the text messages.

As two major risk factors, tobacco smoking and alcohol consumption, are addressed simultaneously for one intervention group, the study could significantly add to the current knowledge of multiple behaviour change interventions [28].

Compared to face-to-face interventions, counselling via Internet and text messaging is economical and matches the lifestyle and communication habits of young people. Furthermore, while the costs of face-to-face counselling interventions significantly increase with an increase in intensity, they do not substantially increase in automated ICT-based interventions, provided the programs have been already developed. Therefore, since integrated intervention approaches are potentially more effective, they could be used with large groups of adolescents and young adults, e.g. in schools or workplaces, with only marginally higher costs compared to the smoking cessation only intervention.

## Abbreviations

HAPA: Health action process approach
ICT: Information and communication technology
MCT: MobileCoach tobacco
MCT+: Mobile coach tobacco+.
